# Fungal Melanin: What do We Know About Structure?

**DOI:** 10.3389/fmicb.2015.01463

**Published:** 2015-12-22

**Authors:** Joshua D. Nosanchuk, Ruth E. Stark, Arturo Casadevall

**Affiliations:** ^1^Division of Infectious Diseases, Department of Medicine, Albert Einstein College of MedicineBronx, NY, USA; ^2^Microbiology and Immunology, Albert Einstein College of MedicineBronx, NY, USA; ^3^Department of Chemistry and Biochemistry, The Graduate Center, The City College of New York, The City University of New YorkNew York, NY, USA; ^4^Institute for Macromolecular Assemblies, The City University of New YorkNew York, NY, USA; ^5^Department of Molecular Microbiology and Immunology, Johns Hopkins Bloomberg School of Public Health, Johns Hopkins UniversityBaltimore, MD, USA

**Keywords:** melanin, fungi, structure, cryptococcus, fungal virulence

## Abstract

The production of melanin significantly enhances the virulence of many important human pathogenic fungi. Despite fungal melanin’s importance in human disease, as well as melanin’s contribution to the ability of fungi to survive in diverse hostile environments, the structure of melanin remains unsolved. Nevertheless, ongoing research efforts have progressively revealed several notable structural characteristics of this enigmatic pigment, which will be the focus of this review. These compositional and organizational insights could further our ability to develop novel therapeutic approaches to combat fungal disease and enhance our understanding of how melanin is inserted into the cell wall.

## Introduction

Melanins rank as one of the great natural pigments as they are synthesized by members of all biological kingdoms, including a wide array of fungi, bacteria, and helminths that cause disease in humans (Nosanchuk and Casadevall, 2003b). Melanins are polymerized from phenolic and/or indolic compounds forming negatively charged, hydrophobic pigments of high molecular weight (White, 1958). We have previously reviewed the broad contributions of melanin to fungal pathogenesis (Gomez and Nosanchuk, 2003; Nosanchuk and Casadevall, 2003b, 2006), which includes melanin’s capacity to alter cytokine responses, decrease phagocytosis, and reduce the toxicity of microbicidal peptides, reactive oxygen species, and antifungal drugs as well as to play a significant role in fungal cell wall mechanical strength.

Despite the profound impact of melanin on fungal diseases as well as the abundance of the polymer in the world’s biomass, the structure of melanin remains poorly defined. Classical biophysical methodologies cannot be applied to decipher the structure of melanin because this polymer is insoluble in aqueous or organic fluids and any attempt at solubilization disrupts its structure. Although melanins have ordered local structures their long-range organization is amorphous, and consequently their structures cannot be solved by X-ray crystallography. Melanins are typically dark in color (usually black or brown), acid resistant and bleached by oxidizing agents (Nicholaus et al., 1964; Prota, 1992; Butler and Day, 1998). The inability to define melanin based on solution-state or crystallographic techniques has prompted the use of alternative approaches to their structural characterization, including electron paramagnetic resonance (EPR) spectroscopy that capitalizes on the presence of a stable organic free radical signature (Enochs et al., 1993).

Melanin in fungi, bacteria and helminths is produced via the polyketide synthase pathway or catalyzed by phenoloxidases [reviewed in (Wheeler and Bell, 1988)]. Melanins formed by the polyketide synthase pathway are called dihydroxynaphthalene [DHN] melanins. A variety of diverse enzymes, including phenoloxidases, tyrosinases, catecholases, and laccases, can generate melanins. Mammalian melanin is synthesized by a tyrosinase (Sanchez-Ferrer et al., 1995). Eumelanin formation is catalyzed by phenoloxidases from DOPA substrates. We have utilized *Cryptococcus neoformans* extensively in our studies of fungal melanin because this yeast-like fungus requires the addition of exogenous phenolic substrates to form eumelanin via laccase, and thus melanization can be closely observed by altering the quantity and type of substrate. Notably, disruption of genes essential for melanin production in *C. neoformans* results in both a reduction in fungal dissemination (Noverr et al., 2004) and lethality (Salas et al., 1996) in murine infection models. Similarly, disruption of genes associated with melanin synthesis in *Aspergillus fumigatus* results in attenuation (Heinekamp et al., 2012). Moreover, we have previously shown that chemical or antibody inhibition of *C. neoformans* melanization results in a reduction in fungal burden and prolonged survival in a murine cryptococcosis model (Nosanchuk et al., 2001; Rosas et al., 2001).

Since melanin is believed to contribute to fungal virulence by reducing the pathogen’s susceptibility to killing by host antimicrobial mechanisms and by influencing the host immune response to infection, melanin and melanin synthesis pathways are indeed potential targets for antimicrobial drug discovery. Hence, a deeper understanding of melanin structure will facilitate the identification of innovative approaches to target this enigmatic polymer. This review discusses our current knowledge on the structure of fungal melanin.

## Fungal Melanin and the Cell Wall

The fungal cell wall is a complex and dynamic construct of branched polysaccharides (particularly β-linked glucans), mannoproteins and proteins (Nimrichter et al., 2005; Latge and Beauvais, 2014). Fungal melanin is typically located within the cell wall, but the distribution and quantity varies widely between species. *C. neoformans* melanin is first detectable along the plasma membrane and fills throughout the cell wall over time (Nosanchuk and Casadevall, 2003b). In contrast, melanin can be found along the outer regions of the cell wall and/or clustered on the cell wall surface of several other pathogenic fungi, including *Candida albicans* (Morris-Jones et al., 2005; Walker et al., 2010), *Aspergillus* sp. (Rosas et al., 2000b; Bayry et al., 2014), *Sporothrix schenckii* (Morris-Jones et al., 2003), *Fonsecaea pedrosoi* (Franzen et al., 1999, 2006), *Paracoccidioides brasiliensis* (Gómez et al., 2001; Taborda et al., 2008), *Coccidioides* sp. (Nosanchuk et al., 2007), and *Histoplasma capsulatum* (Nosanchuk et al., 2002). These cited publications and other reports utilizing electron microscopic techniques have indicated that the layers or clusters of melanin are formed by granules of the polymer.

The most detailed study of fungal melanin localization was achieved using a combination of scanning electron and atomic force microscopy of melanin extracted from *C. neoformans* (i.e., melanin “ghosts”) (Eisenman et al., 2005). This work revealed that cryptococcal melanin is formed by a complex of different sized spherical particles ranging from 40 to 130 nm in diameter. The ovoid nature of the particles was consistent with that reported for eumelanin produced by the cuttlefish *Sepia officianalis* (Clancy and Simon, 2001) and supportive of the granules observed in melanin in *Hortaea werneckii* (Kogej et al., 2007) and *F. pedrosoi* (Franzen et al., 2006). Additionally, the particulate nature of this organization suggested a mechanism by which macromolecules can pass through the melanin, which appears in many images as an impenetrable layer. In fact, studies using size exclusion methods or nuclear magnetic resonance cryoporometry have revealed that there are pores in melanin layers (Eisenman et al., 2005; Jacobson and Ikeda, 2005). The NMR cryoporometry results indicated pores that were mainly 1–4 nm in diameter and less commonly ∼30 nm (Eisenman et al., 2005). Significantly, pore sizes become smaller with increased cell age consistent with increased amounts of melanin deposited in the cell wall.

The pore formation is also significant as these pathways provide a mechanism for macromolecular transport in terms of the formation of layers of melanin rather than a single, uniform polymeric mass. Transmission electron microscopy of thin cross-sections of fungi, especially *C. neoformans*, revealed layers of melanin within the cell wall, with individual layers that are similar in dimension to that of individual melanin particles (Eisenman et al., 2005). X-ray diffraction studies using purified fungal melanins have confirmed the presence and further elucidated details of these layers (Casadevall et al., 2012). For instance, X-ray diffraction revealed a consistent presence of a basic stacked planar sheet structure in melanins isolated from *C. neoformans, Wangiella dermatitidis*, *A. niger*, and *Coprinus comatus* (a common mushroom), and these data were similar to those observed studying other natural melanins. Interestingly, stacking differences varied, such that the stacking distance of melanin layers in *Sepia officinalis* (cuttlefish) melanin was 3.46 Å compared to 4.15 Å for *W. dermatitidis* or 4.45 Å for *A. niger*, and *C. neoformans* stacking was 4.39 Å. The differences in stacking in these fungi may be due to subtle variations in composition as well as differences in the other structures within their individual cell walls. Nevertheless, this consistent finding of layering suggests that X-ray diffraction may be an additional means to define melanins. Moreover, it further raises the question of whether cell wall constituents influence the deposition and organization of melanin in fungi.

## Fungal Vesicles: “Melanosomes”

The discovery of fungal vesicle transport through the complex fungal cell (Rodrigues et al., 2007) provided an explanation for melanin deposition within the cell wall. Several studies have now shown that diverse fungi produce heterogeneous extracellular vesicles that contain lipids, carbohydrates and proteins (Rodrigues et al., 2007, 2008, 2014; Albuquerque et al., 2008, 2012; Eisenman et al., 2009; Oliveira et al., 2009, 2010; Panepinto et al., 2009; Kmetzsch et al., 2011; Rizzo et al., 2014; Peres da Silva et al., 2015; Vargas et al., 2015), many of which are associated with fungal virulence. To reach the extracellular space, intracellularly synthesized macromolecules are targeted to the cell surface for release to the extracellular milieu (Wickner and Schekman, 2005). Notably, vesicle secretion enhances cryptococcal virulence in a murine disease model (Panepinto et al., 2009). However, these vesicles can also be captured within the cell wall. Laccase is a component of *C. neoformans* vesicles (Rodrigues et al., 2008) and vesicle melanization has been confirmed (Eisenman et al., 2009). Although there were variations in vesicle size, a population of melanized vesicles was observed with comparable diameters to those measured in *C. neoformans* melanin (Eisenman et al., 2009). Fungal melanosomes were subsequently described in *C. albicans* (Walker et al., 2010). Hence, it appears that laccase-loaded vesicles can be methodically trapped within the cell wall where they form into layers of melanin.

In combination with these observations, there is now overwhelming evidence from several independent groups that fungal melanization occurs in a specialized vesicle that is analogous to the mammalian melanosome (Franzen et al., 2008; Eisenman et al., 2009; Walker et al., 2010). In hindsight, the need for melanization in a vesicle in *C. neoformans* is obvious because the reaction is catalyzed by a single enzyme that generates a plethora of highly reactive, toxic intermediates that self-react to create melanin. Melanization in vesicles explains much of the biology of fungal melanin: morphology of relatively uniform microspheres with dimensions similar to those of extracellular vesicles (resulting from synthesis therein), the presence of aliphatic components in melanin ghosts resulting from early steps of synthesis in vesicles [described below and in (Zhong et al., 2008)], and budding through melanin (Nosanchuk and Casadevall, 2003a), wherein these vesicles can be simply displaced laterally for the daughter cell to emerge.

## Chitin: A Melanin Anchor

The mechanism(s) for localizing laccase-loaded vesicles to the cell wall have not been resolved; however, several studies suggest that chitin is a primary effector for melanin deposition within the fungal cell wall. Chitin is a long-chain polymer comprised by subunits of β(1,4)-linked N-acetylglucosamine, which is commonly cross-linked to diverse cell wall proteins and polysaccharides. The molecular composition of specific forms of chitin can affect intramolecular and intermolecular interactions of lipid bilayers (Fang et al., 2001), which may facilitate chitin-vesicle engagement. In 1970, Bull reported that melanin was “associated particularly with the chitin” in cell wall fractions of *A. nidulans* (Bull, 1970). This first recognition of the interplay between chitin and melanization in *A. nidulans* has been followed by additional findings in other fungi consistent with the importance of chitin-melanin interactions. For example, deletion of the chitin synthesis WdCHS4 gene in the black fungus *Exophilia (Wangiella) dermatitidis* resulted in a significantly reduced ability to deposit melanin within the cell wall as demonstrated by the accumulation of extracellular pigment (Wang et al., 1999). Mutations of *C. neoformans* chitin synthases, chitin regulatory genes and chitin deacetylases (Banks et al., 2005; Walton et al., 2005; Baker et al., 2007) impede melanization of the cell wall with concomitant detection of melanin in the medium and agar. Additionally, the inhibition of chitinases by methylxanthines results in a ‘leaky melanin’ phenotype in *C. neoformans* (Tsirilakis et al., 2012). *C. albicans* produces granular melanin (Morris-Jones et al., 2005), and deletion of chitin synthase inhibits melanization along the cell wall with concomitant accumulation of melanin particles within the yeast cells (Walker et al., 2010). The effects of defects in chitin that result in either secretion of melanin in *E. dermatitidis* and *C. neoformans* or accumulation of melanin granules in *C. albicans* are consistent with the requirement for vesicle interaction with the chitin. It is noteworthy that an intimate association between chitin and melanin has been described in marine invertebrates (Hwang et al., 2013) as well as insects (Stavenga et al., 2012), suggesting that this scheme for the anchoring of these macromolecular structures extends to other species.

It is highly probable that additional diverse constituents are involved in the localization and maintenance of melanin within the complex cell wall structure. For instance, comparative analyses of *F. pedrosoi* cells cultivated with or without the DHN melanin-specific inhibitor tricyclazole (5-methyl-1,2,4-triazol[3,4] benzothiazole) indicated that melanin was involved in cross-linking diverse cell wall compounds (Franzen et al., 2006).

## Microanalytical Characteristics of Fungal Melanin

As melanin is insoluble, information on melanin structure gleaned in the prior century derives largely from spectroscopic analyses of melanin and characterization of melanin degradation products (Wakamatsu and Ito, 2002). HPLC microanalysis approaches have been particularly useful in the characterization of both pheomelanin and eumelanins (Wakamatsu and Ito, 2002; Wakamatsu et al., 2002). For instance, oxidation of melanized *C. neoformans* cells from cultures or infected mice revealed that both melanins contained PTCA and PDCA (Williamson et al., 1998; Liu et al., 1999), which indicates that cryptococcal melanin is formed of 3,4-dihydroxyphenylalanine (DOPA) oligomers or polymers.

Antibodies (Rosas et al., 2000a; Youngchim et al., 2004; Urán et al., 2011) and peptide ligands (Nosanchuk et al., 1999) have been generated to fungal melanin. Immunofluorescence studies utilizing some of these reagents reveal that there is diffuse, homogeneous binding along the surface of melanins isolated from different fungi, which suggests that there are conserved repeating units serving as epitopes to react with these reagents. Additionally, the peptides that bind melanin are highly positively charged (Nosanchuk et al., 1999), consistent with the finding that melanin is negatively charged (Nosanchuk and Casadevall, 1997; Eisenman et al., 2007; Frases et al., 2011). Moreover, the most reactive melanin-binding peptides are comprised of several aromatic amino acids (Nosanchuk et al., 1999), suggesting that similar aromatic and positively charged structures are present on melanin.

## Recent Insights from New Analytic Approaches

The molecular structure of fungal melanin remains unknown, but significant insights have recently been obtained using advanced nuclear magnetic resonance (NMR) and imaging techniques. By exploiting the requirement of *C. neoformans* for exogenous phenolic substrates to form melanin, pigments generated using natural ^12^C or stable-isotope enriched ^13^C forms of L-DOPA were subjected to high-resolution solid-state magic-angle spinning (MAS) ^13^C NMR to reveal a rich assortment of chemical bonding patterns consistent with alkane, alkene, alcohol, ester, and indole functional groups (Tian et al., 2003). These initial insights have been pursued with site-specific ^13^C-enriched substrates to deduce that developing fungal melanins incorporate additional non-L-DOPA constituents, such as aliphatic groups consistent with triglycerides or phospholipids, and that these are components capable of facilitating interactions between melanin and structures within the fungal cell wall (Zhong et al., 2008). Notably, this latter result is in full accord with the above-mentioned proposal that melanin forms a spatially expanded rather than a discrete layer within the cell wall.

These findings of aromatic and aliphatic structures were followed by a detailed examination of the cell wall and pigment architecture in *C. neoformans* melanin using 2D ^13^C-^13^C correlation solid-state NMR methods (Chatterjee et al., 2015). Consistent with the concept of cell wall constituents comprising a scaffold for the pigment, NMR analyses revealed that the aliphatic moieties of *C. neoformans* melanin included polysaccharide and chitin constituents. It is notable that the chemically resistant melanized *C. neoformans* cell walls exhibit a plethora of proximal and bonded ^13^C-^13^C pairs comprising an aliphatic scaffold consisting of an intimately associated composite of glucan, chitin, mannan, mannoprotein, and phospholipid. In fact, the NMR data support complex architectural networks that include uncyclized aliphatic structures, closely interacting indole-indole pairs, and covalently bound pyrrole-chitin pairs. Moreover, during melanin synthesis, our spectroscopic evidence indicates an early (by day 4) aliphatic scaffold that subsequently incorporates the aromatic components (by day 14). This process is in accord with the increase in negative cell charge (Nosanchuk and Casadevall, 1997; Eisenman et al., 2007; Frases et al., 2011) and reduction in porosity (Eisenman et al., 2005) that occur during aging in a melanizing *C. neoformans* yeast cell.

Fundamental structural differences among melanins derived from L-DOPA, methyl-L-DOPA, epinephrine, and norepinephrine precursors have been demonstrated by ^13^C and ^1^H MAS NMR (Chatterjee et al., 2012). For example, the melanins generated with epinephrine and norepinephrine are observed as thinner by TEM (Garcia-Rivera et al., 2005) and MAS NMR revealed that these melanins also have lower aromatic-to-aliphatic ratios than the more robust melanins formed from L-DOPA and methyl-L-DOPA (Chatterjee et al., 2012). Additionally, the MAS NMR data showed that the EPR signal used historically to define melanin is correlated with the presence of prominent aromatic resonances and that the negative charge of the polymer can be associated with the presence of polar oxygenated aliphatic molecular structures (Chatterjee et al., 2012).

High-field, two-dimensional NMR of ^13^C- and ^15^N-enriched materials was further used to demonstrate that both fungal melanin and synthetic eumelanins share a common indole-based aromatic core (Chatterjee et al., 2014). This investigation provides new information about the supramolecular organization of melanin. For example, Double Cross Polarization and Proton Assisted Insensitive Nuclear Cross Polarization (PAIN-CP) NMR revealed four magnetically distinct indole-like ^13^C-^15^N pairs (Chatterjee et al., 2014) that suggest multiple modes of polymeric assembly involving DHICA and DHI building blocks. The formation of heterogeneous oligomers and polymers is also consistent with assembly of melanins via multiple polymerization pathways.

## Conclusion

**FIGURE 1:**
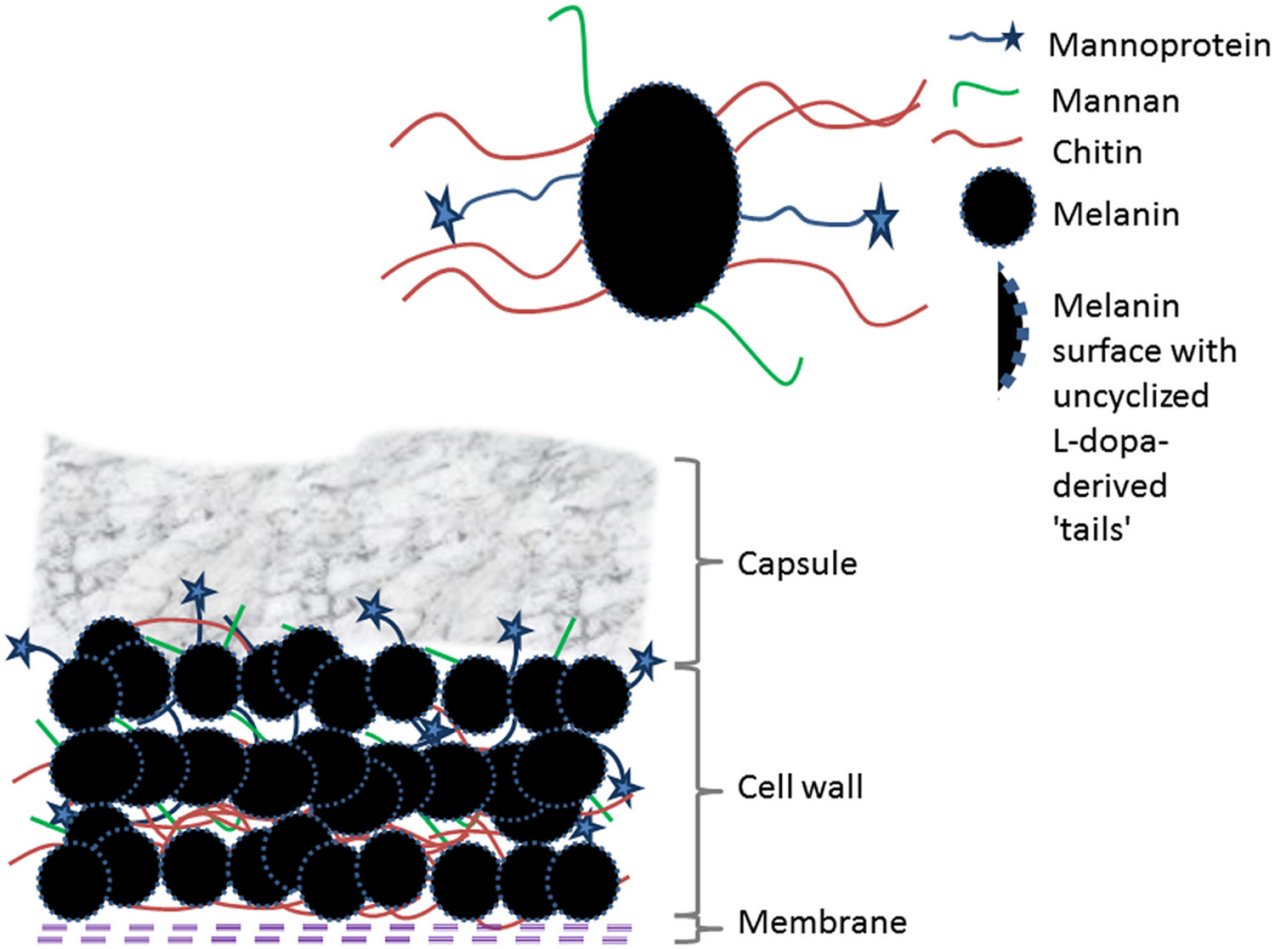
**A conceptualization of melanin organization and identified interactions with macromolecular structures in the cell wall of *Cryptococcus neoformans***.

Although the molecular structure of fungal melanin remains enigmatic, significant progress has been made in understanding particular aspects of its macro- and microstructure during the past 20 years. A representation of our current view of melanin in *C. neoformans* is summarized in **Figure 1**.The increased interest in the structure of melanin has been driven in large part by a remarkable increase in the incidence of diseases due to melanotic fungi. The identification of melanosomes has opened up rich avenues for research that have expanded our appreciation of localization and production of cell wall melanin. The application of NMR techniques has revealed that a chemically resistant aliphatic matrix is assembled prior to significant deposition of indole-based pigments, showing that cell wall composites could serve as a supporting scaffold that fosters eumelanin buildup and presenting opportunities to map out this framework as well as define interlayer stacking interactions and melanin-cell wall interactions. All together, these advances provide a broad platform to gain new insights leading to innovative approaches to combat fungal diseases in which melanin plays a role in pathogenesis. Moreover, these findings can translate into enhanced ability to combat pigment disorders such as melanoma, respond to environmental disasters such as radioactive spills, and generate novel therapeutics such as melanin nanoparticles to ameliorate radiation injury.

## Author Contributions

JN, RS, and AC contributed equally.

## Conflict of Interest Statement

The authors declare that the research was conducted in the absence of any commercial or financial relationships that could be construed as a potential conflict of interest.
